# A Systematic Review of Dietary Supplements in Alzheimer’s Disease

**DOI:** 10.7759/cureus.33982

**Published:** 2023-01-19

**Authors:** Anil Kumar Chimakurthy, Sivani Lingam, Sai Kumar Reddy Pasya, Brian J Copeland

**Affiliations:** 1 Neurology, Barrow Neurological Institute, Phoenix, USA; 2 Neurology, University of Kansas Medical Center, Kansas City, USA; 3 Neurology, Life Neuro Vascular Institute, Guntur, IND; 4 Neurology/Internal Medicine, Osmania Medical College, Hyderabad, IND; 5 Neurology, Louisiana State University Health Sciences Center, New Orleans, USA

**Keywords:** cognitive domains, dementia, alzheimer's disease, memory, dietary supplements, dietary supplements beneficial in alzheimer's disease

## Abstract

Alzheimer’s is the most common neurodegenerative disease among the aging population, which has been a major global challenge. The pathogenesis of the disease is still undetermined but postulated to be involved in various mechanisms including oxidative stress, excitotoxicity, inflammation, cell death, genetic factors, protein accumulation, and degradation. There are few Food and Drug Administration (FDA)-approved drugs available for the treatment of Alzheimer’s disease (AD) that have limited benefits along with associated adverse effects. A retrospective review of randomized double-blind controlled trials of various supplements used in AD patients was performed on a PubMed search from January 1983 to March 2022. We found 10 articles that have shown positive outcomes in various cognitive domains. We conclude that there should be a global standard to endorse the quality and safety of these supplements.

## Introduction and background

Alzheimer’s disease (AD) is the most common neurodegenerative disease, accounting for around 80% of dementia cases, especially in adults greater than 60 years of age [1]. A systematic analysis of the global burden of disease found that, in 2016, about 43.8 million people suffered from AD worldwide [2]. It is estimated that more than 131 million people will suffer from AD by the year 2050, thus making it a major global health challenge [3]. AD is also a growing public health concern. The World Alzheimer’s Report showed that, in 2016 alone, the annual social and economic cost of dementia amounted to US$818 billion [4]. Most forms of dementia, including AD, are more common among females. On average, a 65-year-old woman has a 12% lifetime risk of developing AD, and an overall dementia lifetime risk of 19% [5]. For men, the same figures are 6.3% and 10.9% respectively.

AD is characterized by significant memory, cognitive, and motor impairment. Its etiology and pathophysiology have not been completely elucidated. Epidemiological studies have shown that nearly 90% of AD cases are sporadic, most commonly involving multiple genetic and environmental risk factors, which are generally associated with late-onset forms of the disease [6]. Less commonly, mutations in amyloid precursor protein (APP) and presenilin 1/2, which code for key proteins involved in AD pathogenesis, typically lead to early-onset AD [7]. Despite diverse risk factors and clinical presentations, all forms of AD share several common histologic hallmarks, the most prominent being the extracellular amyloid beta (AB) plaques in various brain regions, especially the medial frontal, lateral temporal and parietal cortices, as well as the intraneuronal accumulation of neurofibrillary tangles, composed of hyperphosphorylated tau, a microtubule-associated protein [8].

**Figure 1:**
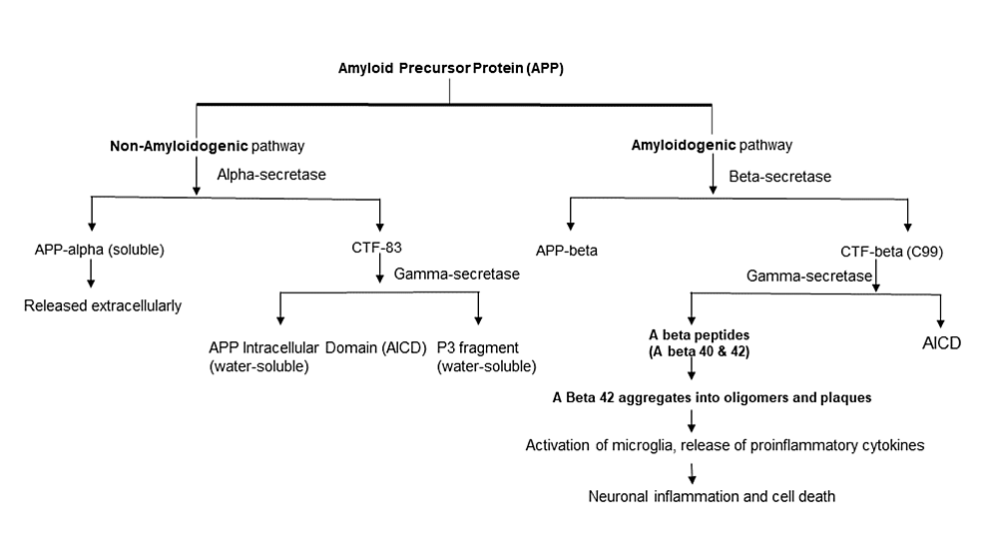
Enzymatic protein breakdown and proposed cellular mechanism involved in Alzheimer’s disease Figure 1 was created based on our understanding of the APP breakdown. CTF - carboxy-terminal fragment, APP - amyloid precursor protein, AICD - APP intracellular domain

AB is derived from APP, a protein normally found on the neuronal cell membrane and thought to play a role in synapse formation and function. The APP is generally degraded by a non-amyloidogenic pathway in which it is cleaved by alpha-secretase, forming soluble APP alpha (which is released extracellularly) and carboxy-terminal fragment (CTF-83) (includes the C-terminal end of the protein and remains membrane-bound). CTF-83 is further cleaved by gamma-secretase, forming APP intracellular domain (AICD) and the P3 fragment, both water-soluble. In contrast, APP in endosomes enters the amyloidogenic pathway, in which beta-secretase cleaves the APP extracellular domain, producing CTF beta (C99) and APP beta. C99 is then cleaved by gamma-secretase to form Abeta peptides (Abeta 40 and Abeta 42) and AICD (Figure 1) [9,10]. Once generated, Abeta 42 is highly prone to aggregation, first into oligomers and then into larger aggregates (plaques). AB oligomers trigger activation of surrounding microglia, which release proinflammatory cytokines, such as IL-1, IL-6, IL-8, TNF alpha, and macrophage inflammatory protein-1. These mediators activate astrocytes, which release various other chemokines and acute-phase proteins and further activate microglial cells. This self-amplifying cycle involving the activation of astrocytes and microglial cells is responsible for the neuroinflammation and neuronal death is seen in AD [11].

Tau, a microtubule-associated protein present in axons, also plays a central role in AD pathogenesis. In AD, tau becomes abnormally hyperphosphorylated, detaches itself from microtubules, misfolds, and forms insoluble aggregates called paired helical filaments (PHFs). These PHFs further condense to form neurofibrillary tangles (NFTs), and tangle burden correlates positively with the extent of neuronal cell death. NFTs may also be responsible for the further propagation of pathology by spreading from cell to cell in a prion-like manner [12]. Several protein kinases are involved in the physiologic regulation of tau phosphorylation, which is crucial to maintaining the protein’s structure and protein. Glycogen synthase kinase 3b (GSK3b) is one of the most active Ser/Thr tau kinases. p25-mediated Cdk5 kinase and mitogen-activated protein kinase (MAPK) also actively regulate Tau phosphorylation [13].

**Figure 2:**
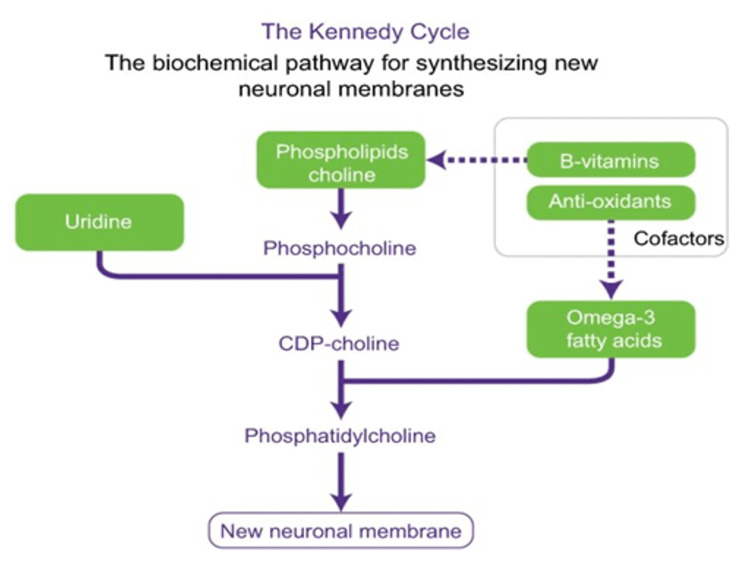
Kennedy cycle Kennedy cycle: The biochemical pathway for synthesizing new neuronal membranes. Developed from Kennedy et al. 1956 [16] and adapted with permission of Annual Reviews from use of phosphatide precursors to promote synaptogenesis, Wurtman RJ, Cansev M, Sakamoto T, Ulus IH, 29, 2009; permission conveyed through Copyright Clearance Center, Inc. [14].

In addition to amyloid plaques and NFTs, another hallmark of early stage-AD is profound synaptic loss, the degree of which often reflects the severity of the memory impairment seen early in the disease. Impaired synthesis of the phospholipids required for neuronal cell membrane and synapse formation and maintenance is thought to play a major role in contributing to the progressive loss of synapses. Certain nutrients such as Omega-3-polyunsaturated fatty acids, docosahexaenoic acid (DHA), uridine, choline, folate, vitamin B12, vitamin B6, vitamin E, vitamin C, and selenium [14,15] are vital for synthesizing these phospholipids (Figure 2) [16]. Studies have found that patients with AD, despite a normal diet, have lower levels of several of these nutrients [17-20].

Due to a gradual decrease in cognitive capacity patients have difficulty with performing activities of daily living with functional limitations. This functional distress and lack of proper treatment for the prevention of disease progression has made researchers go in search of potential treatments for the same. There is no cure for AD, but treatment mainly aims at symptoms and preventing the progression. Currently, Food and Drug Administration (FDA)-approved drugs for AD are donepezil, galantamine, rivastigmine, memantine, and donepezil + memantine for treating cognitive symptoms. Aducanumab is approved by the FDA for the treatment of mild cognitive impairment and mild dementia stages of AD.

Given the critical role of these nutrients in maintaining neural health and synaptic integrity and their proven inadequacy in AD, as well as the lack of disease-modifying therapies available for the disease, many studies have evaluated the possible benefits of supplementing these nutrients in AD. There are some studies that show the beneficial role of nutrients on memory and aging in animal models but failed to produce similar results in human subjects [21]. In this paper, we have sought to provide a systematic review and summary of the most significant results obtained by the major trials conducted to date involving nutrient supplementation in AD.

Methods

**Figure 3:**
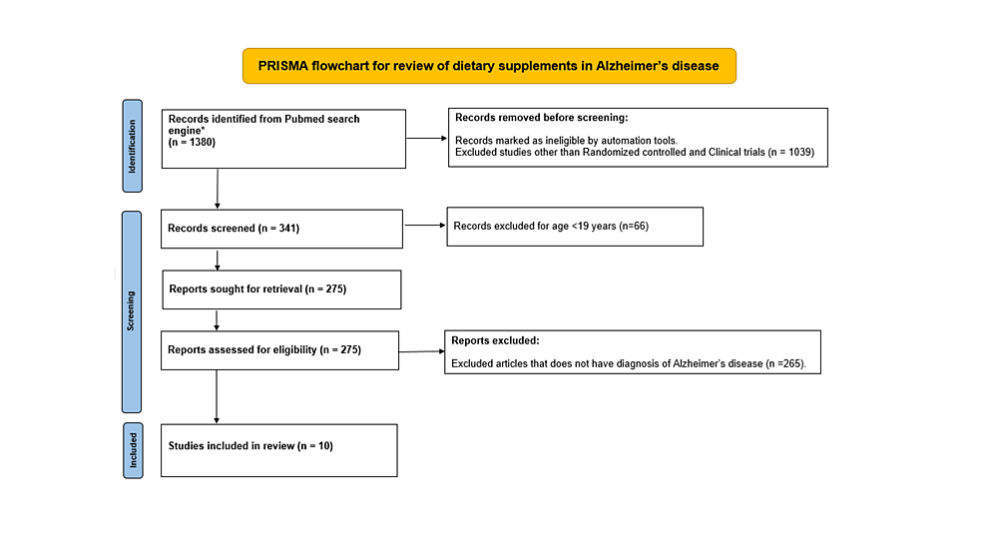
PRISMA flowchart describing screening and selection of articles associated with dietary supplements in Alzheimer's disease *Search limited to Pubmed database between 1984 and March 2022. From: Page MJ, McKenzie JE, Bossuyt PM, Boutron I, Hoffmann TC, Mulrow CD, et al. The PRISMA 2020 statement: an updated guideline for reporting systematic reviews. BMJ 2021;372:n71. doi: 10.1136/bmj.n71

We performed an initial search on PubMed with the search terms dietary supplements and memory during the period from 1983 to March 2022. This search yielded in (n=1380). We have screened with help of automation tools, limiting our search to clinical trials and randomized controlled trials resulting in (n=341). Later the search is limited to adult participants only with age ≥ 19 years (n=275). Articles without a diagnosis of AD and negative outcomes were excluded (n=265), finally, a total of n=10 articles were reviewed in detail (Figure 3).

## Review

The pilot studies reported by Moré et al. described a blend of 100 mg phosphatidylserine (PS) and 80 mg phosphatidic acid (PA) produced from soy lecithin. This randomized, placebo-controlled trial discusses two studies. The first study talks about how the PS+PA combination showed significant improvement in the Wechsler Memory Scale (WMS) scale and the List of Depression symptoms (LDS) scale compared to the placebo. The other study showed a positive influence on pre-post comparison of differences in Activities of Daily Life (ADL) and changes in deterioration, stability, and improvement. Functioning elderly who scored higher on the WMS scale initially showed more significant memory improvements than their counterparts, emphasizing the need for early intervention of these supplements in AD pathology [22]. PS reduces oxidative stress and stimulates neurotransmitter release [23-25].

Another research article by Sun et al. establishes the relationship between hyperhomocysteinemia and malnutrition in AD using the Mini Nutritional Assessment - Short Form (MNA-SF) and how betaine could be a potential treatment option for this disease [26]. It acts by remethylation of homocysteine (HCY) and reduces its effects as HCY impairs DNA repair in hippocampal neurons, making them more prone to damage by Abeta accumulation in AD [27]. Betaine also upregulates memory-related proteins NR1 and NR2A, synaptic proteins, and downregulates inflammatory mediators. Alzheimer’s Disease Assessment Scale-Cognitive subscale (ADAS-cog) analysis used in this study showed significant improvement in recall of words, sino-spatial capacity, and recognition of dual words. Several limitations in the study, such as the small sample size and unclear molecular mechanism, establish the need for further research in this field.

A randomized controlled trial by Andrieu is unique in that it combined a nutritional supplement with a multidomain lifestyle intervention to assess the benefits of polyunsaturated fatty acids supplements on the cognitive decline [28]. The results showed that omega-3 fatty acids alone or in combination with multidomain intervention did not significantly reduce cognitive decline over a three-year study period. But supplementation with DHA alone on participants with age-related cognitive decline showed that it had beneficial effects on visuospatial learning, episodic memory, and verbal recognition memory [29]. This study also showed that those participants with usual risk factors for cognitive declines, such as apolipoprotein E4 (APOE4), Clinical Dementia Rating (CDR) of 0.5 initial ratings, and abnormal amyloid scans showed greater cognitive decline than the inverse. This study showed a positive association between low DHA and EPA concentrations and brain atrophy that has been established as reported in the WHIMS-MRI study [30,31]. As the results are misleading and did not reach significance, there seems to be a need for further research into the benefits of polyunsaturated fatty acids in cognitive decline.

Based on the positive effects of soy isoflavones on cognitively healthy older individuals in several domains [32], Gleason et al. evaluated whether soy isoflavones might improve cognitively impaired adults with a randomized controlled trial comparing isoflavones comprising 85% daidzin and genistin vs placebo [33]. These isoflavones mitigate the negative effects of beta-amyloid on learning and memory performance [34,35]. Assessment of plasma equol levels showed no significant improvement in global cognition using various testing measures; however, there seemed to be a positive correlation between plasma equol levels and speeded dexterity, verbal fluency test in people who were able to metabolize daidzin and genistin. Due to the smaller sample size and multiple outcomes, the effectiveness of soy isoflavones is unclear but may warrant further investigation.

Nolan et al. conducted yet another study to find out the potential benefits of xanthophyll carotenoids and omega-3 fatty acids combined in AD progression [36]. To date, omega-3 fatty acids such as DHA and eicosapentaenoic acid (EPA) seem to be the most promising nutritional intervention in the prevention of AD. Several studies have already proven that omega-3 fatty acids are associated with better cognitive performance [37] and decreased risk of dementia [38]. The levels of xanthophyll carotenoids such as lutein (L), zeaxanthin (Z), and meso-zeaxanthin (MZ) are positively correlated with cognitive performance in healthy individuals [39] and impaired individuals [40]. This preliminary report showed that carotenoids are well absorbed and had a more positive benefit on the maintenance of cognitive and visual function during the interventional period when given in combination with omega-3 fatty acids. This might be due to the fact that, as carotenoids are hydrophobic, these oils help in increasing the uptake and distribution of carotenoids to target tissues. This combination reported functional benefits in memory, mood, and sight. As there are several limitations in this preliminary study, there is a need to focus on larger interventional studies using this combination in the treatment of AD.

Stein et al. conducted a randomized controlled trial of high-dose vitamin D2 followed by intranasal insulin in AD [41]. This was based on the hypothesis that patients with dementia have reduced 25-OH vitamin D levels and it correlates with their cognitive function [42]. Also, because vitamin D may increase insulin receptor expression [43], it was hypothesized that these two could work synergistically to improve cognition and memory in AD patients. Several measures were assessed in this randomized controlled trial, such as the ADAS-cog, Disability Assessment in Dementia (DAD), and Wechsler Memory Scale-Revised Logical memory (WMS-R LM) to check for cognitive improvement. However, this study found no significant positive benefit of adding high-dose vitamin D to ongoing low-dose vitamin D supplementation. Also, intranasal insulin showed no benefit acutely or over 48 hours. The ADAS-cog score did not change significantly during the 16-week low-dose vitamin D intervention in the placebo group of AD patients. Therefore, the beneficial effect of low-dose vitamin D in AD cannot be completely excluded.

Scheltens et al. conducted two phases of trials using the Souvenaid. Souvenaid contains Fortasyn™ Connect (Nutricia; Zoetermeer, the Netherlands), which is a combination of precursors and other specific nutrients required to enhance neuronal membrane formation. Fortasyn™ Connect includes ingredients that are required for synapse formation to potentially reverse or stop synaptic loss, a hallmark feature of AD. Souvenaid I showed that Souvenaid is well tolerated and improved 12-week memory performance measured by delayed verbal recall testing [44, 45] without any biomarkers for assessment of synaptic activity. Souvenaid II was conducted to further evaluate the effects of Souvenaid on memory in the early stages of AD. Neuropsychological test battery (NTB) score is used rather than ADAS-cog for better assessment of memory in early AD. The primary outcome assessed was the memory cognitive domain Z-score, which showed a significant difference in favor of the active group compared to the placebo. The secondary parameter included electroencephalogram (EEG)-Phase Lag Index (PLI), which is used as a marker of functional connectivity. PLI for the delta band of EEG also showed a significant difference in favor of the active group suggesting new synapse formation with Souvenaid. These results thus necessitate the need for further investigation on a large scale [46].

The positive results of the Souvenaid studies in Alzheimer's led to the LiPiDiDiet trial, a major, multicenter randomized controlled trial studying the effects of Fortasyn™ Connect. The primary outcomes of the study, such as the NTB composite Z score, showed no significant difference from the placebo group. However, several secondary outcomes were found to be significant in favor of Fortasyn™ Connect. Those include a lesser deterioration in Clinical Dementia Rating Scale-Sum of Boxes (CDR-SB), less reduction in hippocampal volumes, and less increase in ventricular volume on brain imaging. The specific cognitive outcomes that showed significant benefits include attention, memory, and executive function [47]. These findings along with those from other studies [48-50] suggest a possible benefit of Fortasyn™ Connect in treating the early stages of AD.

To examine the benefit of early nutritional interventions in AD, Remington et al. conducted a randomized controlled trial of a nutraceutical formulation (NF) for individuals with AD vs placebo for a period of 3/6 months. NF contained a combination of folate, alpha-tocopherol, vitamin B12, S-adenosyl methionine, N-acetylcysteine, and acetyl-L-carnitine. The primary outcome of interest was a cognitive performance on the Clox-1 and Dementia Rating Scale-2 (DRS-2). Secondary outcomes of behavioral and psychological symptoms of dementia (BPSD) and ADL were assessed using the Neuropsychiatric Inventory (NPI) and Alzheimer’s Disease Cooperative Study-Activities of Daily Living Scale (ADCS-ADL) scores. Participants receiving NF showed statistically significant improvement in primary outcomes within the first three months compared to placebo. There was also a 1.86-fold improvement in total NPI scores vs placebo, though not statistically significant. No effect was seen on ADL in either group. It was also concluded that NF had more impact on those with mild or moderate dementia than severe dementia and was also found to be more effective when started in earlier stages of AD [51].

Following the positive findings in the randomized controlled trial, an open-label extension of 24 participants with AD was continued for one year. Cognitive assessments again included Clox-1 and DRS-2. Secondary outcomes of BPSD and ADL were assessed using NPI and ADCS-ADL scores. No significant changes were seen in the participants at any time point when compared to participants who started NF de novo; however, scores also did not decrease over the course of the study. Although a very small sample size, the relative stability in cognitive performance and BPSD in addition to maintaining ADL for as long as 28 months supports the notion that nutritional supplements may be beneficial in AD (Table 1) [52].

**Table 1:** Results of a retrospective review of dietary supplement that has a beneficial role in various cognitive domains in patients' with Alzheimer's disease ADAS-Cog - Alzheimer’s Disease Assessment Scale-Cognitive Subscale, ADCS-ADL - Alzheimer’s Disease Cooperative Study-Activities of Daily Living Subscale, ADL - Activities of Daily Living, BPI - Brief Pain Inventory, BPSD - Behavioral and Psychological Symptoms of Dementia, CERAD - Consortium to Establish a Registry for Alzheimer’s Disease, CDR-SB - Clinical Dementia Rating Scale-Sum of Boxes, DAD - Disability Assessment in Dementia, DRS-2 - Dementia Rating Scale, FFQ - Food Frequency Questionnaire, GDS - Geriatric Depression Scale, MMSE - Mini-Mental State Examination, NPI - Neuropsychiatric Inventory, NTB - Neuropsychological Test Battery, PLI - Phase Lag Index, WMS - Wechsler Memory Scale, WMS-R - Wechsler Memory Scale-Revised, WMS-RLM - Wechsler Memory Scale-Revised Logical Memory

Study	No	Supplement	Markers and outcomes	Domains improved
1. Moré MI et al. [22]	Study 1: n= 72 (60-80) years. Study 2: n= 96 (50-90) years.	Phosphatidylserine (PS) and Phosphatidic acid (PA)	Study 1: At 3 months - WMS. Study 2: At 2 months - General self-reporting, scoring of ADL are assessed.	Study 1: Positive influence on memory and mood in pre-post comparison, significant(p<0.05). Study 2: Positive influence on daily functioning, positive trends on emotional state, self-reported general condition. Significant(p<0.05).
2. Sun J et al. [26]	n=97 (74.6 +/- 9.2) years.	Betaine, a zwitterionic quaternary ammonium compound (200 microgram/kg).	-Homocysteine levels - Tau Phosphorylation and phosphatase activity -memory-related and synaptic protein levels - ADAS-Cog analysis.	-Homocysteine level significantly elevated in malnourished (p<0.05) - Homocysteine levels and malnourishment results in AD - Betaine able to reduce hyperphosphorylation of Tau protein and increase phosphatase activity (P<0.05) -Betaine increases memory-related protein (NR1 & NR2A) (P<0.05) and synaptic proteins - Betaine cause a significant improvement in recall of words, visuospatial capacity, recognition of dual words.
3. Andrieu S et al. [28]	n=1680 Age>70 years.	Docosahexaenoic acid 800mg Eicosapentaenoic acid 225mg In Addition to multidomain intervention, omega 3 polyunsaturated fatty acids, placebo.	Composite Z-Score at 36 months.	Neutral Study. The multidomain intervention plus supplement showed improvement in composite Z-score which is not statistically significant.
4. Gleason CE et al. [33]	n= 65 (> 60 years).	Purified soy isoflavones (mainly Daidzin and Genistin) capsules; 100 mg/day.	Assessed at 3 & 6 months Study subjects were assessed for weekly dietary intake using FFQ. Cognitive domains specifically assessed include visual-spatial memory, visual-motor, verbal fluency, and dexterity. Isoflavone assays (Equol) were also measured. Grooved Pegboard; dominant and non-dominant hand.	Isoflavone increased significantly at 3 and 6 months compared to the placebo. Total plasma equol levels significantly correlated with a measure of speeded dexterity. Plasma equol levels and verbal fluency performance showed a positive association. Phonemic fluency has a positive correlation r=0.29, p=0.04.
5. Nolan JM et al. [36]	n=40 patients Trial 1, n=12 Trial 2, n=13 Trial 3, n=15 normal controls.	Trial/Formulation 1: xanthophyll carotenoids only. Trial/formulation 2: xanthophyll carotenoids along with fish oil.	Trials 1, 2, 3 measured serum conc. Of lutein (L) and meso zeaxanthin (MZ) at baseline and after 6 months after intervention In trial 2 LPC 22:6 and LPC 20:5 were measured at baseline and at 6 months to gauge DHA and EPA response respectively.	Patients on (Formulation 2) over 18 months show less Progression of AD compared (Formulation 1). 1. Clinical progression of AD was less in T2 compared to T1 (P=.003) 2. Patient carer reported functional benefits in memory, sight, and mood in T2 patients.
6. Stein MS et al. [41]	n=63 (age >60).	Open labeled, 3000 IU vitamin D2 for 8 weeks. Followed by Humulin-R (100IU/ml) - nasal insulin	Baseline assessment - ADAS-cog and DAD, Folstein MMSE, WMS-RLM; Serum calcium, albumin, creatinine, uric acid. Primary endpoint - ADAS-Cog, WMS-RLM immediate and delayed scores. Secondary endpoint-ADAS-cog word recall, word recognition; GDS, BPI.	Neutral study. ADAS-cog improved by a median (IR) of 9 with nasal insulin after placebo high-dose vitamin D (p = 0.02) but may represent regression to the mean as WLS-R LM did not change. Intranasal insulin benefits may be restricted to early disease.
7. Scheltens P et al. [44,46]	n=259 Mean age 73.8 (51-89)	Daily 125ml(125kcal) Nutricia Souvenaid - has Fortasyn^TM^ Connect composed of: Eicosapentaenoic acid (EPA), 300mg, Docosahexaenoic acid (DHA), 1200mg, Phospholipids 106mg Choline 400mg Uridine monophosphate 625mg Vitamin E (alpha-tocopherol equivalents) 40mg, Vitamin C 80mg, Selenium 60µg, Vitamin B12 3µg Vitamin B6 1µg, Folic acid 400µg	The primary outcome assessed includes the memory function domain (NTB z score). Secondary outcomes-Executive function domain score. - using WMS-r Digit span, Category fluency, Trail making part A and B tests, Controlled oral word association test. PLI of various bands alpha, beta, theta, delta is analyzed on EEG. PLI marker of functional connectivity. Blood tests (Homocysteine levels, Vit E, RBC EPA, and DHA) to test for adherence.	-Improved NTB composite Z score at 24 weeks (p = 0.023; Cohen’s d = 0.21; 95% confidence interval [−0.06]–[0.49]). -No significant intervention effect on the NTB executive function domain. -Connectivity analysis (PLI) for the delta band revealed a significant in favor of the active group -increased erythrocyte DHA and EPA, plasma vitamin E, and decreased plasma homocysteine were detected in the active group, reported high compliance at 24 weeks.
8. Soininen H et al. [47]	n=311 (prodromal AD patients) Age not defined but participants have a diagnosis of AD.	Souvenaid, a daily supplement that contains Fortasyn^TM^ Connect as the active ingredient composed of: Eicosapentaenoic acid, 300mg Docosahexaenoic acid, 1200mg Phospholipids 106mg Choline 400mg Uridine monophosphate 625mg Vitamin E (alpha-tocopherol equivalents) 40mg Vitamin C 80mg Selenium 60µg, Vitamin B12 3µg Vitamin B6 1µg, Folic acid 400µg	NTB composite Z-score over 24 months NTB Z-score was based on CERAD 10-word list learning immediate recall, CERAD 10-word delayed recall, CERAD 10-word recognition, category fluency, and the Letter Digit Substitution test.	IN NTB composite Z-score statistically significant findings include -CDR-SB -MRI total hippocampal volume reduction -MRI ventricular volume reduction. Fortasyn^TM^ Connect has positive outcomes on attention, memory, and executive function when considered separately.
9. Remington R et al. [51]	n=106 Aged 77.8 ± 8.4 years.	NF-2 Tabs/day dose composed of: folic acid 400µg Vitamin B12 6ug alpha-tocopherol 30IU, S-Adenosylmethionine SAM 400mg (200 mg active iron) N-Acetyl Cysteine 600mg Acetyl-L-Carnitine 500mg Placebo-identical in appearance but inert.	Primary outcome-Cognitive outcome determined by Clox-1 and Age- and Education-Corrected Mayo’s Older Americans Normative Studies Scaled Score (AEMSS) of DRS-2 Secondary outcomes- BPSD and daily function determined by NPI and ADCS-ADL.	Improved Cognitive performance, memory at 3 months and improved with statistical significance (Clox-1 p = 0.0083, 95% CI [0.4481, 2.9343]; DRS-2 p = 0.0266, 95% CI [0.1722, 2.7171]). BPSD improved NPI improved 1.86-fold in NF group NF more effective in earlier stages of AD.
10. Remington R et al. [52]	n=24. Aged 78.4 +/- 5.7 years.	Nutraceutical formulation (NF) composed of: folic acid 400µg Vitamin B12 6ug alpha-tocopherol 30IU, S-Adenosylmethionine SAM 400mg (200 mg active iron) N-Acetyl Cysteine 600mg Acetyl-L-Carnitine 500mg prepared by Nutricap Labs.	Primary outcome-Cognitive performance determined by Clox-1 and DRS-2 Secondary outcomes-BPSD and daily function determined by NPI and ADCS-ADL.	-baseline, 3 months for 12 months. (Participants already on NF prior to study not de novo, so not improved). Participants did not display any significant or clinical change in cognitive performance of BPSD score over the course of 12 months. Crossover of placebo to NF showed improvement or maintenance paralleling There is no decrease in cognitive performance or BPSD over the course of 12 months observed in Placebo.

## Conclusions

In our retrospective review of dietary supplements, all were randomized controlled trials demonstrating an improvement in self-reported or physician-tested measures of severity, demonstrating the possible benefit of supplementation. Some of these supplements are used in addition to anticholinesterase inhibitors, which exhibit a beneficial role. Given the heterogeneity of purification, marketing, and labeling standards for supplements and nutraceuticals depending on country and provider, a more uniform standard needs to be adopted to optimize the quality and safety of these supplements. Some studies were limited by their small sample size and exclusion of comorbid conditions. Future cross-sectional studies incorporating a large, diverse sample population as well as longitudinal studies with participants in different stages of AD are needed to better evaluate the role of these supplements in improving clinical outcomes. There remains a lack of literature on whether nutrient supplementation in healthy young adults with a family history of AD or other risk factors can delay the onset or progression of the disease. Large prospective longitudinal studies of this group are needed to determine whether these supplements can be used for primary prevention. Further studies are also needed to evaluate the potential for long-term systemic toxicity of these supplements, as well as their interactions with current FDA-approved treatments for AD and other commonly prescribed medications in patients with AD patients. Given the established dangers of polypharmacy in elderly patients, identifying the potential drug-drug interactions of these supplements will allow for more rational prescribing.
